# Juvenile Smokers

**Published:** 1892-05-14

**Authors:** 


### Passing Topics.

### JUVENILE SMOKERS.

We fear that the Manchester gentleman who upon the score of injury to health has appealed to the London newspapers to embark upon a crusade against the practice of smoking by juveniles, and to invoke Parliamentary interference, would hardly carry his point, though he succeeded in enlisting the whole power of the press. We could well wish it were otherwise, and that the effort he is making gave greater promise of the desired result. But legislation could at most prevent smoking by boys in public places, and an enactment of that kind, though it would remove a part of what is undoubtedly a nuisance and a scandal, and would to that extent be welcome, must prove at best inoperative in regard to health unless Parliament is prepared to go the length of requiring the production of a certificate of age by purchasers of tobacco.

Secret smoking and smoking indoors would necessarily be worse in its effects upon the physical and moral well-being of growing lads than smoking in the open air, and the medical protests which are sensible and well-timed at the present would become more emphatic and even less useful than if things remained as they are. The alarming increase of juvenile smoking is concurrent with the decline of parental control and home discipline, and it is one more sign that the tendency of the age we live in is subversive of many old-fashioned notions of order and propriety. Time was when a youth in his teens would have thought twice before helping himself to a cigar in the company of his elders, and if he had indulged his imitative faculty at all in the matter of smoke, it would have been in secret session with certain choice spirits of his own years, and with a full consciousness of the punishment which would follow upon discovery.

No doubt mere vanity is accountable for much of the public smoking by youths. We recollect how the first cigar we ourselves could venture upon without fear of immediate and disastrous consequences was displayed to the world from the top of an omnibus as a proud and visible sign of assured manhood, and, as we believed, to the admiration of large numbers of interested observers of both sexes. But at all events this was after arrival at legal maturity, the sense of deference and decorum in those days being sufficiently robust to prevent mere children from venturing publicly upon an experiment of the kind.

All is changed; parents, whatever their private opinions may be, think no course but submission is open to them, and forbear even to protest. Daily scholars fling away cigarette ends as they reach the school precincts, and the outside seats of omnibuses and the smoking carriages of suburban railway trains are crowded, morning and evening, with office-boys and very junior clerks, cigar or pipe in mouth, looking supremely ridiculous, and, of course, profoundly ignorant of those conditions of life which render smoking a solace to their elders, and, when not immoderately indulged, a benefit. It is a question calling for grave reflection, and if legislation can effect anything, by all means let the subject be pressed upon the attention of Parliament, but on the whole we should prefer that the whipping the young rascals deserve should be administered at home or at school rather than by the hands of a public functionary. Agricola.

